# "Attending to Collaboration" in Major System Change in Healthcare in England: A Response: Comment on "‘Attending to History’ in Major System Change in Healthcare in England: Specialist Cancer Surgery Service Reconfiguration"

**DOI:** 10.34172/ijhpm.2022.7661

**Published:** 2022-12-11

**Authors:** Alec Fraser, Lorelei Jones, Colin Lorne, Ellen Stewart

**Affiliations:** ^1^King’s Business School, King’s College London, London, UK.; ^2^School of Medical and Health Sciences, Bangor University, Bangor, UK.; ^3^School of Social Sciences and Global Studies, Faculty of Arts and Social Sciences, The Open University, Milton Keynes, UK.; ^4^Department of Social Work and Social Policy, University of Strathclyde, Glasgow, UK.

**Keywords:** Major System Change, Healthcare Reconfiguration, Regional Reforms, Collaborative Governance

## Abstract

In this short article we comment upon the recent article by Perry et al "Attending to History" in Major System Change in Healthcare in England: Specialist Cancer Surgery Service Reconfiguration. We welcome the engagement with power, history and heuristics in the Perry et al paper. Our article discusses the importance of researcher positionality in Major System Change research, alongside managerial power and the centrality of politics to remaking health and care services. Additionally, we highlight the work of Ansell and Gash focused on ‘collaborative governance’ and its potential to offer insight in relation to Major System Change.

## Introduction

 We are grateful to the editors of *International Journal of Health Policy and Management* for inviting us to comment on Perry and colleagues’ stimulating and timely article^1^ that emphasises the importance of “attending to history” in Major System Change in healthcare in England. We find that the article has much to offer and builds on a valuable and impressive body of work on Major System Change in the National Health Service (NHS) produced by these authors and others over many years.

 The article engages with one of the five simple ‘rules’ proposed by a highly influential heuristic from Best et al^2^: in this case, the instruction to “attend to history.” As researchers, we all desire our work to be of value and influence for those using and working within healthcare systems.^3^ As such, the clarity and simplicity of heuristics such as that developed by Best et al^2^ has real merit. At the same time, Perry et al^1^ identify the risks of simplifying inherently complex and contested events. Ongoing testing and questioning of such heuristics or frameworks will, we hope, make them more suitable for the complexity of contemporary health and care systems.

 The explicit reference to power in relation to Major System Change by Perry et al^1^ is one example of how the Major System Change literature is maturing. Power has too often been overlooked in studies of regional service reconfigurations in the NHS and internationally,^4^ and Perry et al^1^ are to be commended for exploring the issue in this article.

 Temporal (and spatial) elements of policymaking have also been frequently downplayed in Major System Change studies so their engagement with the work of Suddaby and Foster^5^ is also welcome. This acknowledges some of the subjective and relational aspects of “history,” rather than a simple recounting of unproblematic historical events, providing a number of useful analytical windows through which to develop their aforementioned interest in power.

## Positionality, Power and Politics

 Notwithstanding our high regard for the article, we wish to indicate some of the missed opportunities we identify therein. Firstly, while the methods used in the article are robust and impressive, there is a notable absence of discussion regarding researcher positionality. Longitudinal in-depth qualitative enquiry drawing on non-participant observations, key stakeholder interviews and extensive documentary analyses offer an excellent multifaceted approach to deep understanding within and across complex healthcare settings.^6^ However, this depth and range of coverage requires some consideration of how, and with what expectations, the researchers had access in this case. There appears to be limited reflection on the roles played by the research team in the research process and of the fact that they are producing a powerful ‘history’ of the reconfiguration themselves. To what extent are the histories presented in the article those of the informants or the researchers?

 Researcher positionality in the work of evaluation is important in both relation to how we interpret policy and in situating our findings and recommendations but this is downplayed in the article. Paying careful attention to how health researchers are themselves entangled as intermediaries within the policies they are studying may generate important insights into how policy ‘successes’ or ‘failures’ are identified, co-created, learnt from and circulated.^7,8^ Whilst acknowledging word-count constraints and conventional assumptions about what ‘fits’ or is seen as ‘appropriate’ in terms of the expectations of conventional methods sections in Health Services Research, we feel it might have been helpful had the methods section of the paper provided more detail about issues of researcher reflexivity. It might also have been good to learn more about how organisational documents were interrogated and folded into the analysis.

 Connectedly, whilst welcoming the explicit recognition of the importance of power in the interpretation of Major System Change in the article, we feel the article would have been strengthened further by deeper and more consistent engagement with the workings of power. The article discusses ‘discursive power’ and issues of ‘framing’ in the later sections, but we feel the analysis could be strengthened by a more thorough and diverse exploration of power modalities in social science theory as applied to health policy.^9^

 Perry et al^1^ usefully identify and describe some of the key political tactics and strategies used by actors with managerial responsibilities to minimise dissent from professional actors. We know such conflicts can frequently delay and disrupt efforts towards Major System Change, and the article highlights the effectiveness of managerial power over professionals through these exclusionary authoritative actions. However, the article is relatively silent on the broader politics of the reorganisation. Whilst the reconfiguration process will no doubt have included some kind of consultation with wider publics, the article does not refer to this, nor does it shed much light on the actions and views of local politicians nor the wider shifting balance of political forces such as the impacts of central government-imposed ‘austerity’ shaping the logics of consolidation of services despite references to ‘political pressures.’ This may lead to a picture of reconfiguration that prioritises a managerial view of the world, focused on minimising dissent, thereby displacing other struggles and contestations associated with reorganising healthcare services. By extension, the article further legitimises the evasion of politics, dialogue and consent in Major System Change by reinforcing the idea of reconfiguration as a technical exercise.^4^

 We turn now to our second critique of the article. One of us was also researching Major System Change in Greater Manchester at a similar time to the Perry et al^1^ research team so we feel we can offer a complementary and hopefully relevant view of the reorganising of health services in Greater Manchester as part of the wider remaking of the city-region. Perry et al^1^ use three out of four of Suddaby and Foster’s^5^ varieties of history: history as *fact*, history as *power*, and history as *sensemaking*, but they do not use the fourth variety – history as *rhetoric*. It seems to us that an engagement with history as rhetoric might have added a further critical edge to the article and that some of the broader narratives within the article feel somewhat divorced from temporality. This criticism is linked to our earlier observation about depoliticization and cultural-political work to ‘take the politics out’ of health service reconfiguration.

 A lot of work and effort has gone into reassembling health and care in Greater Manchester into a seemingly coherent, integrated system working together ‘as one.’^10^ Certainly, claims of an entrepreneurial city-region working collaboratively and consensually builds on the careful nurturing of rhetorical politics about the remaking of Greater Manchester – with the city of Manchester at its political and economic heart – over many decades. And, Greater Manchester’s health and social care ‘devolution deal’ indeed became the latest example of how the performing of a pragmatic ‘togetherness’ was used in attempts to leverage new public resources and private investment into the city-region.^11^ This history as rhetoric has been important to the ‘invented tradition’^5,12^ of devolution. It is significant that Major System Change in relation to Oesophago-Gastric Cancer Services was often used to support the wider case for devolution, and paradoxically, devolution was also given as an example of how Major System Change would be made to work. We suggest a more critical engagement with the history of collaboration, devolution and Major System Change might have added a deeper, more politically sensitive account of change and policymaking in times of austerity.

## Attending to Collaboration

 The missed opportunities described above relate, we suggest, to how our approaches to studying change need to keep pace with an increasingly complex landscape characterised by interdependency and collaboration. The Best et al^2^ ‘simple rules’ heuristic or framework, and indeed the concept of ‘Major System Change,’ invokes top-down mandated change, out of step with an era of ‘systems and creativity.’^13^ Health systems are increasingly turning to collaborative efforts to plan public services for localities, often in recognition of the limitations of managerialism, particularly downstream implementation failures.^14^ Collaborative strategies also recognise that stakeholders, including patients, communities, and staff, can make a valuable contribution to innovation that can be sustained over time.^15^

 Practitioners and researchers need new approaches to understand the collaborative process and the necessary conditions for success. Social science theory is a rich source of insights on the social, cultural, and political dimensions of collaborative planning and management. An example is Ansell and Gash’s model of collaborative governance which discerns the key influences on the collaborative process, and which includes many of the relational aspects touched on in Perry and colleagues’^1^ analysis. Grounded in 137 case studies, the model highlights the role of facilitative leadership in repairing relationships and rebuilding trust, including all relevant stakeholders, and establishing procedural legitimacy.^14^ At the heart of the collaborative process is a commitment to meaningful inclusion in decision-making and a willingness to understand and appreciate the interests and perspectives of other stakeholders. No ‘simple rules,’ but useful knowledge to sustain collective working to address long-standing challenges in health and social care.

 Finally, we suggest that a new age of improved and more trusting collaboration raises questions for the health policy research community. Indeed, the present moment – with the shift to integrated care systems in the English NHS, alongside calls for ever greater collaboration across many health systems around the world – is a good time to analyse and respond to this. What should our roles as health and care policy researchers be in relation to reorganising services and systems? Should we be offering lessons, critique, or distilling rules about effective change? If so, to which communities, how and why, and through what types of media? There will be many diverse and conflicting responses to these questions perhaps reflecting the tensions between commitments to criticality and utility^16^ in the work that we as health and care policy researchers collectively undertake. It is in this spirit, that we invite researchers to reflect on and openly engage with these issues and attend to collaboration as well as attending to history.

## Ethical issues

 Not applicable.

## Competing interests

 Authors declare that they have no competing interests.

## Authors’ contributions

 All authors discussed the key ideas for the article. AF wrote a first draft based on these discussions. LJ, CL, and ES commented on the first draft and edited and added further text to produce an agreed final text.
